# Combined application of melatonin and *Bacillus* sp. strain IPR-4 ameliorates drought stress tolerance via hormonal, antioxidant, and physiomolecular signaling in soybean

**DOI:** 10.3389/fpls.2024.1274964

**Published:** 2024-06-21

**Authors:** Odongkara Peter, Muhammad Imran, Shifa Shaffique, Sang-Mo Kang, Nkulu Kabange Rolly, Chebitok Felistus, Saqib Bilal, Zhao Dan-Dan, Md. Injamum-Ul-Hoque, Eun-Hae Kwon, Mohammad Nazree Mong, Ho-Jun Gam, Won-Chan- Kim, In-Jung Lee

**Affiliations:** ^1^ Department of Applied Biosciences, Kyungpook National University, Daegu, Republic of Korea; ^2^ Biosafety Division, National Institute of Agricultural Sciences, Rural Development Administration, Jeonju, Republic of Korea; ^3^ Center for International Development, Kyungpook National University, Daegu, Republic of Korea; ^4^ Natural and Medical Science Research Center, University of Nizwa, Nizwa, Oman; ^5^ Crop Foundation Research Division, National Institute of Crop Sciences, Rural Development Administration, Wonju, Republic of Korea

**Keywords:** *Bacillus* sp., melatonin, drought, stress tolerance, soybean, antioxidants

## Abstract

The role of melatonin and plant growth-promoting rhizobacteria (PGPR) in enhancing abiotic stress tolerance has been widely investigated. However, the mechanism underlying the interaction between melatonin and PGPR in drought stress tolerance is poorly understood. In this study, we investigated the role of *Bacillus* sp. strain IPR-4 co-inoculated with melatonin (IPR-4/MET) to ameliorate drought stress response in soybean. Initially, 16 random isolates were selected from a previously pooled collection of isolates from soil at plant physiology lab, and were screesn for plant growth promoting (PGP) traits and their survival rate polyethylene glycol (PEG6000) (5%, 10%, and 15%). Among these isolate Bacillus sp. strain IPR-4 were selected on base of its significant PGP traits such as the survival rate gradient concentrations of PEG6000 (5%, 10%, and 15%) compared to other isolates, and produced high levels of indole-3-acetic acid and organic acids, coupled with exopolysaccharide, siderophores, and phosphate solubilization under drought stress. The Bacillus sp. strain IPR-4 were then validated using 16S rRNA sequencing. To further investigate the growth-promoting ability of the *Bacillus* sp. IPR-4 and its potential interaction with MET, the bacterial inoculum (40 mL of 4.5 × 10^−8^ cells/mL) was applied alone or in combination with MET to soybean plants for 5 days. Then, pre-inoculated soybean plants were subjected to drought stress conditions for 9 days by withholding water under greenhouse conditions. Furthermore, when IPR-4/MET was applied to plants subjected to drought stress, a significant increase in plant height (33.3%) and biomass (fresh weight) was observed. Similarly, total chlorophyll content increased by 37.1%, whereas the activity of peroxidase, catalase, ascorbate peroxidase, superoxide dismutase, and glutathione reductase increased by 38.4%, 34.14%, 76.8%, 69.8%, and 31.6%, respectively. Moreover, the hydrogen peroxide content and malondialdehyde decreased by 37.3% and 30% in drought-stressed plants treated with IPR-4 and melatonin. Regarding the 2,2-diphenyl-1-picrylhydrazyl activity and total phenolic content, shows 38% and 49.6% increase, respectively. Likewise, *Bacillus–*melatonin-treated plants enhanced the uptake of magnesium, calcium, and potassium by 31.2%, 50.7%, and 30.5%, respectively. Under the same conditions, the salicylic acid content increased by 29.1%, whereas a decreasing abscisic acid content (25.5%) was observed. The expression levels of *GmNCED3*, *GmDREB2*, and *GmbZIP1* were recorded as the lowest. However, *Bacillus*–melatonin-treated plants recorded the highest expression levels (upregulated) of *GmCYP707A1* and *GmCYP707A2*, *GmPAL2.1*, and *GmERD1* in response to drought stress. In a nutshell, these data confirm that *Bacillus* sp. IPR-4 and melatonin co-inoculation has the highest plant growth-promoting efficiency under both normal and drought stress conditions. *Bacillus* sp. IPR-4/melatonin is therefore proposed as an effective plant growth regulator that optimizes nutrient uptake, modulates redox homeostasis, and enhances drought tolerance in soybean plants.

## 1 Introduction

Soybean *Glycine max* L. is an important crop cultivated worldwide because of its many health benefits and contribution to significant revenue in developed and developing countries (Khojely et al., 2018). The remarkable popularity of soybeans can be attributed to their indispensable presence in food supplements and the growing global appetite for soy-based products. Between 2005 and 2010, soybean production experienced robust annual growth of 2.9%, and this period witnessed a significant expansion of soybean cultivation globally. Looking ahead to the 2020–2030 period, the anticipated annual production rate is expected to further moderate to 1.8% (Qiao et al., 2023). Despite its popularity, soybean production faces challenges worldwide, particularly in dry areas where drought-induced yield reductions of over 50% are common (Fidantemiz et al., 2019). The increasing occurrence of extreme weather conditions such as heavy rainfall, heat waves, and tropical cyclones further threatens global soybean yield and food security (Yadav et al., 2019).

Similar to other abiotic stresses, drought stress has been identified as a major cause of yield reduction in soybean, impeding plant growth and development from germination to maturity (Poudel et al., 2023). Drought episodes reduced soybean yields in Brazil by approximately 11.8% during 2011/2012 (Faé, 2019), 30% in Australia (2017–2020) (Bert et al., 2021), and in the United States during the 2015/2016 cropping seasons (Folger and Cody, 2014). Plants manifest drought effects through a decrease in leaf area index, pod production, seed size, harvest index, and grain quality (protein and oil content) (Bhattacharya, 2021). The other effects of drought stress include the induction of osmotic stress, which may lead to reductions in mineral elements or ions such as hydrogen (H^+^), potassium (K^+^), chlorine (Cl^−^), and nitrate (NO_3_^−^).

To address these challenges, various approaches have been explored, such as the use of organic manures, biotechnology for developing drought-resistant genotypes, and the application of biostimulants and beneficial microbes as biofertilizers, including plant growth-promoting rhizobacteria (PGPR) (Kumari et al., 2019). PGPR are root-associated bacteria that can play beneficial roles in plant growth and development and in biofertilization by enhancing nutrient supply to plants (Backer et al., 2018). The introduction of rhizobacteria is considered a game changer in improving plant growth under extreme climate (Bhat et al., 2023). Rhizobacteria establish a symbiotic interaction between plants, with plants providing shelter and nutrients while benefiting from minerals and organic compounds synthesized by the microbes in the rhizosphere (Manasa et al., 2021). These interactions promote plant growth through mechanisms such as nitrogen fixation phosphate solubilization, production of plant hormones, and antagonism against plant pathogens (Aeron et al., 2020). Microbes play a role in converting minerals such as phosphorus and iron into a form that plants can easily absorb, such as orthophosphates (H_2_PO_4_) and (HPO_4_)^2−^ and ferric ions (Fe^3+^). These microbes can solubilize phosphates and produce iron-chelating compounds, making the minerals readily available for plants to take up (Kour et al., 2019), and previous research suggested using PGPR as a biofertilizer to increase crop yield and reduce the harmful effects of synthetic fertilizers (Etesami and Adl, 2020).

The involvement of PGPR in abiotic stress tolerance mechanisms has been established (Etesami and Maheshwari, 2018). In essence, a major way through which PGPR enhance drought tolerance in plants is by colonizing the rhizosphere or endo-rhizosphere of plants and by producing exopolysaccharides (EPS); phytohormones such as abscisic acid (ABA), salicylic acid (SA), indole-3-acetic acid (IAA), and 1-aminocyclopropane-1-carboxylase deaminase; volatile organic compounds, inducing the accumulation of ionic osmolytes; and antioxidant systems (Vurukonda et al., 2016). Likewise, studies have suggested that PGPR increase shoot and root biomass and enhance photosynthetic efficiency and transcriptional regulation of stress-responsive genes.

When plants are subjected to abiotic stresses, such as drought, they undergo several changes at the physiological, biochemical, and molecular levels, which result in the activation of downstream adaptive response mechanisms to either counteract, avoid, or escape the stress. Under drought stress, plants produce reactive oxygen species (ROS), which serve as signaling molecules that activate the appropriate adaptive response mechanism toward stress tolerance. However, overaccumulation of ROS causes oxidative stress that may lead to oxidative damage and culminate in programmed cell death (Yanık et al., 2020). To negate, plants activate non-enzymatic and enzymatic [ascrobate peroxidase (APX), catalase (CAT), peroxidase (POD), and superoxide dismutase (SOD)] systems that help lower the overaccumulation of ROS and alleviate their negative effects (Majidinia et al., 2017). Likewise, during drought stress, a vast amount of transcriptional reprogramming occurs, causing the induction or suppression of stress-responsive genes, including transcription factors (TFs) (Hrmova and Hussain, 2021). Similarly, plant hormones such as ABA, SA, and IAA are widely known for their roles as plant growth regulators and their involvement in stress signaling events. Among them, ABA prevails over others and is indicated to be the most responsive phytohormone in abiotic stress conditions (Singh and Roychoudhury, 2023).

In recent years, there has been a growing interest in the use of melatonin (MET) as a phytohormone and antioxidant to alleviate drought stress in crops, including soybean. MET is a biogenic indole amine that shares a structural relationship with other compounds such as IAA, tryptophan, and serotonin. MET has also emerged as a potential growth regulator due to its functions in seed germination and plant biomass production, enhancing the photosynthetic efficiency of plants, retarding senescence, and delaying flowering and fruit ripening (Tiwari et al., 2021). Initially discovered in animal species, reports indicate that MET is continuously synthesized in plant organelles and is involved in plant growth and development by enhancing photosynthetic efficiency while interacting with other growth-promoting compounds, enzymes, and calcium (Zhao et al., 2019). The industrial discovery of MET as a phytohormone and antioxidant with major functions as a free radical scavenger has supported efforts to extensively study its application in crop production to alleviate plant abiotic stress such as drought stress (Tiwari et al., 2021) and salt stress (Talaat, 2021). The mechanism employed by MET in plant stress tolerance occurs through modulation of physiological processes and biochemical reactions and by enhancing the transcriptional regulation of MET production proteins (Muhammad et al., 2024). According to Ahmad et al. (2023), MET promotes tolerance to oxidative stress by restoring the oxidation-reduction (redox) equilibrium, which involves respiratory burst oxidative homologs (RBOHs). Similarly, Altaf et al. (2022) reported that MET promotes drought stress tolerance by modulating plant growth, root architecture, photosynthesis, and antioxidant defense systems. MET plays important roles in adaptive response mechanisms toward stress tolerance, including acting as a signaling molecule during stress occurrence, enhancing ROS-scavenging efficiency, safeguarding the photosynthetic system by reducing oxidative stress, and regulating several other physiological processes in plants (Jahan et al., 2023).

Although the potential benefits of using PGPR or melatonin for drought stress tolerance in soybean have been reported, the ability of co-inoculating PGPR with melatonin to enhance abiotic stress tolerance in plants remains unknown. Therefore, this study aimed to investigate the ability of *Bacillus* sp. strain IPR-4 co-inoculated with MET to enhance soybean plant growth and development in response to drought stress. In addition, the potential mechanism underlying changes at the physiological, biochemical, and molecular levels in soybean plants co-inoculated with *Bacillus* sp. IPR-4 and MET in response to drought stress was investigated. Furthermore, this work monitored the transcriptional reprogramming of drought-responsive genes in relation to the co-inoculation of *Bacillus* sp. and MET.

## 2 Materials and methods

### 2.1 Sample collection and *in-vitro* isolation

In the current study, 16 random isolates were selected from a previously pooled collection of isolates (Sahile et al., 2021), sampled from Pohang Beach (longitude 128°378′E and latitude 37°0343′N) in South Korea. These isolates were preserved at the Crop Physiology Laboratory, Kyungpook National University, Daegu, Republic of Korea. They were re-tried for plant growth-promoting (PGP) abilities such as the production of EPS, siderophore, and IAA and the solubilization of phosphate.

### 2.2 Screening for plant growth-promoting parameters

To test for EPS production, 25 g L^–1^ of Luria–Bertani (LB) broth was mixed with 0.33% sucrose, 2% agar, and 0.8 L^−1^ of Congo red dye and then autoclaved. Then, 40 µL of each strain, having a concentration of 10^−8^ cells/mL (OD_600_ nm 0.38), was inoculated on EPS medium and incubated at 28°C for 5 days as described by Kang et al. (2021). For siderophore production, an agar medium was prepared by dissolving 27.216 g L^–1^ of 1,4-piperazinediethanesulfonic acid (PIPES) in 900 mL of distilled water, and the pH was adjusted to 6.5 with sodium hydroxide (NaOH) or hydrochloric acid (HCl). Furthermore, a CAS-blue dye solution was prepared by suspending 60.5 mg of chrome azurol S (CAS) and 72.9 mg of ammonium bromide (NH_4_Br) in 90 mL of distilled water. An iron III (Fe^3+^) solution was prepared by suspending 27 mg of iron (III) chloride (FeCl_3_) in 100 mL of distilled water containing 36 μL of HCl. The Fe^3+^ solution was mixed with 90 mL of the CAS-blue solution, followed by autoclaving separately. Then, after cooling, the CAS-blue solution was added to 900 mL of agar solution containing PIPES and poured onto a plate. Thereafter, 40 µL of each bacterial inoculum with a concentration of 10^−8^ cells/mL was streaked onto CAS-blue medium plates and incubated at 28°C for 5 days. The plates were observed for any orange halo formation to detect siderophore production. Phosphate solubilization was evaluated on the National Botanical Research Institute’s phosphate growth medium (NBRIP) containing 10 g L^−1^ of glucose, 5 g L^−1^ of calcium phosphate [Ca_3_(PO_4_)_2_], 0.1 g L^−1^ of ammonium sulfate [(NH_4_)_2_SO_4_], 0.25 g L^−1^ of magnesium sulfate heptahydrate (MgSO_4_.7H_2_O), 0.2 g L^−1^ of potassium chloride (KCl), 5 g L^−1^ of magnesium chloride hexahydrate (MgCl_2_.6H_2_O), and 10 g L^−1^ of agar. The IAA assay was performed using the Salkowski reagent. All analyses followed the method described by Khan et al. (2014) with slight modifications.

Based on their individual performances, such as EPS and siderophore production, solubilization of phosphate, and formation of IAA, the four isolates (IPR-4, IPR-7, IPR-8, and IPR-9) were selected and further screened for drought tolerance using polyethylene glycol (PEG6000) at four different concentrations (0%, 5%, 10%, and 15%) as follows: 100 μL of each isolate inoculum with a concentration of 10^−8^ cells/mL (OD_600_ nm 0.45) was added to 5 mL of sterilized nutrient broth supplemented with PEG, followed by incubation at 30°C at 150 rpm for 3 days. The absorbance was read at 8-h intervals (8, 16, 24, and 32 h) at OD_600 nm_ using a T60 UV-Vis Spectrophotometer (PG Instruments Ltd., UK) as described by Kim et al. (2020). Based on these results and the bacterial growth pattern under 15% PEG conditions, *Bacillus* sp. strain IPR-4 was selected for downstream experiments.

### 2.3 Molecular identification of *Bacillus* sp. strain IPR-4 using 16S rRNA

To identify *Bacillus* sp. strain IPR-4, genomic DNA was extracted from 200 mL of the isolate culture having a concentration of 10^−8^ cells/mL (OD_600 nm_ 0.4) using a QI Amp DNA Mini kit (Qiagen) according to the manufacturer’s instructions, with a final eluted volume of 100 μL. The DNA was used as a template in a polymerase chain reaction (PCR) using 16S rRNA gene-specific primers: 27F primer (5′-AGAGTTTGATCACTGGCTCAG-3′) and 1492R primer (5′-CGGCTTACCTTGTTACGACTT-3′), as described earlier by Khan et al. (2020). The PCR reaction mixture comprised 1 Ex Taq buffer (Takara Bio Inc., Japan), 0.8 mM of dNTP, 0.02 units μL^−1^ of Ex Taq polymerase, 0.4 mg mL^−1^ of bovine serum albumin (BSA), and 10 pM of each primer. The PCR conditions were as follows: enzyme activation at 94°C for 5 min, initial denaturation at 94°C for 60 s, annealing at 55°C for 40 s, initial extension at 72°C for 60 s in 30 cycles, and final extension at 72°C for 10 min. The amplicons were purified using a Wizard^®^ PCR Preps DNA Purification System (Promega, Madison, WI, USA) and sent for sequencing using a Big Dye Terminator Cycle sequencing kit (Applied Biosystems, Forster City, CA, USA) according to the manufacturer’s instructions. The resulting sequences were aligned using the Chromas Lite 2.1.10.1 (https://technelysium.com.au/wp/chromaspro/2022). The 16S rRNA sequence was submitted to the National Center for Biotechnology Information (NCBI/GenBank nucleotide databases under the accession number OP303352.1). For the 16S region of bacteria, the basic local alignment search tool (BLAST, https://blast.ncbi.nlm.nih.gov/Blast.cgi) was used for sequence homology analysis. To construct a neighboring tree, the ClustalW feature was used in the molecular evolutionary genetics analysis (MEGA 11.0.10) (Department of Biological Sciences, Tokyo Metropolitan University, Tokyo, Japan) (Saitou and Nei, 1987). To statistically support the nodes in the phylogenetic trees, bootstrap (1,000 replications) was performed with the alignment threshold value above 80%, and the strain IPR-4 was identified with 93% similarity (Felsenstein, 1985; Tamura et al., 2004).

### 2.4 Indole-3-acetic acid production ability of *Bacillus* sp. strain IPR-4

The endogenous IAA content was quantified as previously described earlier (Sahile et al., 2021). Briefly, the *Bacillus* sp. strain IPR-4 was grown for 3 days in LB broth. Subsequently, 1.5 mL of culture was centrifuged at 10,000×*g* for 10 min 4°C, and the supernatant was filtered through a 0.2-μm Millipore membrane filter (dismic: 25 cs, Advantec, Tokyo, Japan). Then, 900 μL of each filtrate was reacted with 800 μL of Salkowski reagent. To determine IAA production, gas chromatography–mass spectrophotometry (GC/MS) was used. A 5-day-old culture of an IPR-4 isolate was grown in LB broth supplemented with PEG at four concentrations: 0% (control consisting of nutrient broth), 5%, 10%, and 15%. Five days later, 50 mL of the IPR-4 isolate culture was centrifuged at 6,000×*g* for 10 min. Afterward, 10 mL of the supernatant was acidified to pH 2.8, followed by dilution with an equal amount of ethyl acetate. The upper layer was transferred to a round bottom flask and added with 50 µL (0.5 ng) of IAA internal standard (IAA-d_5_) and vacuum-dried in a rotary evaporator. The residue was washed with 5 mL of 60% methanol (MeOH) and filtered through a C18 column. The obtained solution was then vacuum-dried and diluted with 1 mL of 100% MeOH, sonicated, and dried under nitrogen gas (N_2_). The dried sample was later methylated with 60 µL of diazomethane (CH_2_N_2_), incubated in the dark for 30 min, and redried with N_2_ before injecting 2 µL into GC/MS with a selective ion monitoring (SIM) system (6890N network GC system, and 5973 Network mass selective detector; Agilent Technologies, Palo Alto, CA, USA).

### 2.5 *In-vitro* analysis of organic acid production ability of *Bacillus* sp. strain IPR-4

To quantify organic acid production by the *Bacillus* sp. strain IPR-4, the method described by Khan et al. (2022) was followed with modifications. Briefly, a 5-day-old culture of the IPR-4 strain was grown in LB broth supplemented with PEG at four different concentrations: 0%, (control), 5%, 10%, and 15%. After 5 days, 5 mL of the bacterial culture was centrifuged at 7,000 rpm for 10 min at 4°C, and the supernatant was filtered through a 0.45-µm Millipore membrane filter (dismic: 25 cs, Advantech, Tokyo, Japan). Then, 1 µL of the sample was injected into a high-performance liquid chromatography (HPLC) system (Waters HPLC System-Agilent 1100 series, Agilent ChemStation + software, USA). The following were also used: RSpak KC-811 column (8.0 × 300 mm), eluent 0.1% (w/v) phosphoric acid (H_3_PO_4_) in distilled water (HPLC grade), and flow rate 1.0 mL min^−1^ at 40°C. The retention time for pretreatment and determination by HPLC was 30 min, and the peak areas obtained in the chromatograms were compared with the peaks of standard solutions containing a lyophilized mixture of 0.1 mg mL^–1^ sodium malate (Na_2_(C_2_H_4_O(COO)_2_)), 2.5 mg mL^–1^ sodium citrate (Na_3_C_6_H_5_O_7_), sodium oxalate (Na_2_C_2_O_4_), sodium formate (HCOONa), sodium succinate (C_4_N_4_Na_2_O_4_), and sodium acetate (CH_3_COONa) (Sigma-Aldrich, USA).

### 2.6 Plant experimental treatments of *Bacillus* sp. IPR-4/MET in soybean

#### 2.6.1 Seed treatment and germination

For the current study, Kyungpook National University Soybean Genetic Research Center provided soybean seeds. The seeds were surface-sterilized with 2.5% sodium hypochlorite for 5 min and then washed twice with double-distilled water. The surface-sterilized seeds were then placed in a germination tray containing autoclaved horticulture soil with the following composition: peat moss (13%–18%), perlite (7%–11%), coco peat (63%–68%), and zeolite (6%–8%), with micronutrients NH_4_ 90 mg kg^–1^, NO_3_ 205 mg kg^–1^, P_2_O_5_ 350 mg kg^–1^, and potash (K_2_O) 100 mg kg^–1^ as previously described by Imran et al. (2022). Germination trays were then placed in a growth chamber under the following conditions: temperature 24°C–28°C, 14/10 h light/dark cycles, moisture range 55%–65%, and 1,000 Em^2^ illuminance using fluorescent bulbs. At the VC stage (V1, one unrolled trifoliate leaf), soybean seedlings of uniform size were selected and transplanted into plastic pots (10 cm × 9 cm) containing the same horticulture soil.

#### 2.6.2 Experimental setup and treatments

Experimental plant materials were divided into two groups. The first group (A) included i) control plants (only distilled water) and ii) sole *Bacillus* sp. strain IPR-4 inoculated, iii) sole MET treated, and iv) *Bacillus* sp. IPR-4/MET co-inoculated. The second group (B) included i) sole drought-treated plants, ii) combined *Bacillus* sp. IPR-4 inoculated and drought stress, iii) combined MET and drought-treated plants, and iv) co-inoculated *Bacillus* sp. IPR-4/MET and drought-treated plants. To assess the effects of *Bacillus* sp. IPR-4/MET co-inoculation, a method earlier used by Imran et al. (2021b) was employed. In essence, 40 mL of *Bacillus* sp. IPR-4 culture with a concentration of 10^−8^ cells/mL (OD_600_ nm 0.2) and 50 mL of 100 µM of MET were soil-drenched on each plant in groups A and B for 5 days, and distilled water was provided to control plants in group A. After pretreatment with the isolate IPR-4/MET for 5 days, the water supply was cut off to each plant in group B: drought-treated plants (i) were kept at 30%–35% soil moisture by regularly monitoring soil moisture using a soil moisture meter (Demetra, E.M. System Soil Tester; Tokyo, Japan). Plants in group B (ii, iii, and iv) received *Bacillus* sp. IPR-4 suspension culture, MET solution, and IPR-4/MET solutions, respectively. The experiments followed a complete randomized block design (CRBD) with eight treatments and eight replicates (*n* = 64 plants). At the end of the stress period of 9 days, four plants were sampled from each treatment (*n* = 32 plants) for measurement of growth parameters such as shoot length and root length. Chlorophyll (Chl) contents were measured with a SPAD chlorophyll meter (Konica Minolta, Tokyo, Japan). A total of 32 plants were harvested on day 9 of stress treatment, and the harvested shoot samples consisting of four plants per treatment were dried in liquid nitrogen, macerated, and stored in a refrigerator at −80°C for subsequent trials.

### 2.7 Effects of the isolate IPR-4/MET on soybean growth parameters

#### 2.7.1 Chlorophyll-a and chlorophyll-b measurements

To determine chlorophyll-a and chlorophyll-b contents, the method previously described by Vimala and Poonghuzhali (2015) was used, with slight modifications. Briefly, 0.2 g of freeze-dried shoot samples were mixed with 150 μL of 80% MeOH, and the mixture was centrifuged at 10,000×*g* for 10 min. Next, 200 μL of the supernatant was extracted, and the absorbances of chlorophyll-a and chlorophyll-b were recorded at 470 nm and 645 nm, respectively, using a GO Multiskan Microplate Spectrophotometer (Thermo Fisher Scientific, USA). Chlorophyll content was calculated using the formula by Arnon (1949):

$\text{chlorophyll-a}\left(\text{mg }{\text{g}}^{–1}\text{FW}\right)=\left[\left({\text{R}}_{1}\times {\text{A}}_{470}\right)-\left({\text{R}}_{2}\times {\text{A}}_{645}\right)/1,000\times \text{FW}\right]\times \text{V}$


$$\text{chlorophyll-b}\left(\text{mg }{\text{g}}^{–1}\text{FW}\right)=\left[\left({\text{R}}_{1}\times {\text{A}}_{645}\right)-\left({\text{R}}_{2}\times {\text{A}}_{470}\right)/1,000\times \text{FW}\right]\times \text{V}$$

where R_1_ is reading 1 and R_2_ is reading 2. A represents absorbance, FW represents fresh weight, and V represents the extraction volume.

#### 2.7.2 Reactive oxygen species-scavenging activity assay

The thiobarbituric acid (TBARS) assay was used to determine the degree of lipid peroxidation by changing the MDA content, as described by Jambunathan (2010). Briefly, 0.2 g of freeze-dried shoot samples were mixed with 5 mL of 0.1% trichloroacetic acid (TCA) and centrifuged at 10,000×*g* for 10 min. Then, 100 μL of the supernatant was diluted with 150 μL of 20% TCA and 2 mL of 0.5% 2-thiobarbituric acid (TBA), heated at 95°C for 30 min, and cooled on ice for 10 min. The optical density was measured at 530 nm for reading 1 (R_1_) and 600 nm for reading 2 (R_2_). The final MDA reading was calculated by subtracting reading 2 from reading 1 (R_1_ − R_2_).

For H_2_O_2_ detection, 0.2 g of freeze-dried shoot samples were homogenized with 5 mL of 5% TCA and centrifuged at 10,000×*g* for 10 min. Then, 5 μL of the supernatant was added with 100 μL of potassium iodide (1 mM) followed by 50 μL of phosphate buffer (pH 8.0), and the absorbance was read at 390 nm as described by Imran et al. (2021a) with modifications. At the same time, for detection by diaminobenzidine tetrahydrochloride (DAB) staining, two fresh leaf samples were harvested from control and drought-treated plants at stage V3 and placed in a vessel containing a DAB solution at a concentration of 0.5 mg mL^−1^. The DAB solution was prepared by dissolving DAB in 25 mM Tris–HCl (pH 3.8). To ensure proper staining, the leaves were subjected to vacuum infiltration three times at 100 mbar for 1 min each. Subsequently, the leaves were bleached with 95% ethanol (EtOH) to remove chlorophyll, followed by incubation at 85°C in a water bath for 30 min. The stained leaves were fixed with a solution of ethanol, lactic acid, and glycerol in a 3:1:1 ratio, carefully mounted on a glass slide, and photographed.

#### 2.7.3 Determination of antioxidant enzyme activity

POD levels were determined using the method described by Imran et al. (2021a). Briefly, 0.2 g of freeze-dried shoot samples were dissolved in 5 mL of 0.1 M phosphate buffer (pH 6.8) and centrifuged at 10,000×*g* for 15 min at 2°C, and the obtained 50 μL of supernatant was added to a reaction mixture containing 25 μL of 5% H_2_O_2_ and 100 μL of 100 mM phosphate buffer (pH 5.5) followed by 50 μL of 50 mM pyrogallol. Phosphate buffer (pH 5.5) was used as a negative control in the absence of the enzyme. POD activity was determined as a unit change per minute by measuring the absorbance at 470 nm for 3 min.

The reduced glutathione (GSH) and CAT activities were quantified using a method described earlier (Asaf et al., 2017). Briefly, 0.2 g of freeze-dried shoot samples were homogenized in 5 mL of 5% TCA and centrifuged at 10,000×*g* for 10 min at 4°C, 100 µL of the supernatant was transferred to a 96-well plate, and 50 μL of Ellman’s reagent was added, followed by 100 μL of 150 mM phosphate buffer. The absorbance was recorded at 420 nm with three replicates. For CAT, 0.2 g of the freeze-dried shoot sample was extracted with 5 mL of CAT extraction buffer [50 mM of Tris–HCl, 3 mM of MgCl_2_, 1 mM of ethylenediaminetetraacetic acid (EDTA), and 1% polyvinylpyrrolidone (PvP)] and centrifuged at 10,000×*g* for 10 min at 4°C; 50 μL of the supernatant was added with 100 μL of 10 mM phosphate buffer (pH 6.8) followed by 50 μL of H_2_O_2_; absorbance was recorded at 240 nm.

For APX activity, 100 mg of the shoot sample was extracted with 1 mL of 50 mM phosphate buffer (pH 7.0) containing 1 mM of ascorbic acid and 1 mM of EDTA. After homogenization, the mixture was centrifuged at 4,830×*g* for 15 min at 4°C, 200 μL of the supernatant was taken, and absorbance was recorded at 290 nm as described previously by Imran et al. (2021a).

For quantification of SOD, the method of Mccord and Fridovich (1969) was used with modifications. Briefly, 0.2 g of the freeze-dried shoot sample was extracted with 5 mL of SOD extraction buffer (50 mM of Tris–HCl and 10 mM of EDTA) and centrifuged at 10,000×*g* for 10 min at 4°C, and 50 μL of the supernatant was mixed with 150 µL of extraction buffer followed by 50 μL of pyrogallol in a test well (A). Fifty microliters of the supernatant was added to 200 μL of the extraction buffer in a test well plate (B), and 150 μL of the extraction buffer followed by 100 μL of pyrogallol was added in the test well (C). The SOD absorbance was recorded at 420 nm. SOD activity (%) was calculated using the following formulas:


$$\text{SOD activity}\left(\%\right)=\left[\left(1-\left(\text{A}-\text{B}/\text{C}\right)\right.\right]\times 100$$

where (A) is reading 1, (B) is reading 2, and (C) is reading 3.

#### 2.7.4 Quantification of free radical scavenging

To quantify the free radical scavenging activity of 2-diphenyl-1-picrylhydrazyl (DPPH), the methods previously described by Manzocco et al. (1998) were followed with modifications. Briefly, 0.2 g of the freeze-dried shoot sample was added with 5 mL of 90% ethanol and centrifuged at 10,000×*g* for 10 min at 4°C, 100 μL of the supernatant was added with 100 μL of DPPH reagent, and the absorbance was read at 517 nm. The total phenolic content (TPC) was determined using Folin–Ciocalteu assays as described earlier (Imran et al., 2021a) with slight modifications, using gallic acid as the standard. In brief, 0.2 g of the freeze-dried shoot sample was homogenized with 5 mL of 70% methanol solution containing 2% formic acid and 28% EtOH, ultrasonicated for 30 min, and centrifuged at 10,000×*g* for 10 min at 4°C. Fifty microliters of the supernatant was filtered through a 0.45-μm membrane filter and added with 100 μL of Folin–Ciocalteu reagent, followed by 70 μL of 10% NaCl_3_, and the absorbance was read at 750 nm.

#### 2.7.5 Determination of essential nutrient elements

Inductively coupled plasma mass spectrometry (ICP-MS) was used to determine calcium (Ca), potassium (K), silicon (Si), and magnesium (Mg) uptake in soybean plants treated with drought stress following the method described by Kang et al. (2014). In brief, 0.1 g of the freeze-dried shoot sample was soaked in HCl (0.5 M) and oven-dried. The obtained digested samples were then subjected to analysis using ICP-MS (Optima 7900DV; Perkinelmer, Waltham, MA, USA) to assess the generation of these essential ions under different treatments.

#### 2.7.6 Quantification of endo-melatonin

Endogenous MET was extracted and quantified in soybean plant leaves using the melatonin enzyme-linked immunosorbent assay (ELISA) Kit (Enzo Life Sciences, Farmingdale, NY, USA) according to the manufacturer’s protocol. In brief, two soybean fresh leaves from drought-treated and control plants were rinsed three times with distilled water and wiped with a paper towel; 0.1 g of each leaf sample was ground to a fine powder using liquid nitrogen and homogenized in 125 µL of 1× stabilizer followed by the addition of 750 µL of cold ethyl acetate and vortexed. The mixture was then incubated on ice for 5 min and then centrifuged at 5,000×*g* for 10 min. The organic layer formed was transferred to a fresh microtube and dried under liquid nitrogen gas. The pellets obtained were suspended in 250 µL of 1× stabilizer for further quantification according to the manufacturer’s protocol.

#### 2.7.7 Quantification of endogenous ABA and SA

Endogenous ABA and SA were quantified using the method of Verslues (2017) with some modifications. Briefly, ABA was extracted from 0.3 g of freeze-dried shoot samples, and chromatography was performed using the Me-[2H6]-ABA standard. The fraction was methylated with diazomethane for subsequent detection and quantification of ABA using a GC/MS model (6890N Network GC System, Agilent Technologies). The software from Thermo Quest Corp. (Manchester, UK) was used to monitor signal ions at *m*/*z* 162 and 190 for Me-ABA and *m*/*z* 166 and 194 for Me-[2H6]-ABA. For the quantification of SA, the method described by Khan et al. (2020) was followed. Briefly, 0.3 g of the freeze-dried shoot sample was reacted with 5 mL of 100% MeOH and centrifuged at 10,000×*g* (three times). The pooled methanolic extracts were vacuum-dried and suspended in 2.5 mL of 5% TCA and recentrifuged at 10,000×*g* to collect the supernatant. The aqueous extract was separated using ethyl acetate, cyclopentane, and isopropanol (ratio of 100:99:1, v/v/v) and dried using nitrogen gas, and the SA content was quantified by HPLC−fluorescence detection instrument (Shimadzu RF-10AXL, Kyoto, Japan).

#### 2.7.8 Molecular evaluation of relative gene expression and stress signaling effects of *Bacillus* sp.-IPR-4/MET co-inoculation on soybean under drought stress

RNA was extracted using the methods previously described by Imran et al. (2018). The role of IPR-4/MET in drought-treated soybeans was validated, as well as the expression of drought stress-responsive genes and dehydration response transcription factors such as *DREB2*, *bZIP1*, and *ERD1*, along with biosynthesis and catabolic genes of ABA such as *NCED3*, *CYP707A1*, and *CYP707A2*) and SA biosynthesis gene *GmPAL2.1*. Briefly, 0.1 g of fresh leaf sample was macerated in liquid nitrogen and immediately transferred to an RNase-free E-tube containing the following extraction buffers: 0.05 M of Tris–HCl, pH 7.5; 0.25 M of NaCl; 20 mM of EDTA 8; 4% (w/v) PVP; and 1% (w/v) tricine-sodium dodecyl sulfate (SDS). RNA quality and concentration were measured using Nano-Q (Optizen nano q, Mecasys Co., Ltd. Daejeon, Korea). Subsequently, cDNA synthesis and quantitative real-time PCR (qRT-PCR) were performed as described by Chan et al. (2004). In brief, 1 µg of RNA was used to synthesize cDNA using the BioFACT™ RT kit (BioFACT™, Daejeon, Korea) according to the manufacturer’s instructions. The synthesized cDNA was used as a template for further evaluation of transcript accumulation using qRT-PCR (Eco™ Illumina™, California, CA, USA). A detailed list of the genes and their corresponding primers are shown in **Supplementary Table 1**. For qRT-PCR, a 2× real-time PCR Master Mix (BioFACT™) was added to 10 µM of each gene-specific primer and 100 ng of template cDNA with a final volume of 20 µL, under a two-step 40 cycles of PCR.

### 2.8 Statistical analysis

The experimental treatments were performed independently in triplicate. One-way ANOVA in MS Excel was used to assess the significant difference between the treatment means. Duncan’s multiple range test (DMRT) was used to determine critical values for comparison between the mean of treatments at a significance level of *p ≤*0.05. The Statistical Analysis System (SAS 9.1) was used for DMRT analysis. For the graphical representation, we used GraphPad Prism software (version 9.5.1, San Diego, CA, USA).

## 3 Results

### 3.1 Isolation, screening for PGP traits, and identification of the IPR-4 isolate

The ability of the 16 isolates to produce exopolysaccharides, siderophores, and solubilize phosphates was examined. The results revealed that 10 isolates, namely, IPR-1, IPR-2, IPR-4, IPR-6, IPR-8, IPR-9, IPR-10, IPR-12, IPR-13, and IPR-15, showed positive test results for the production of EPS from glucose in the Congo red medium, leading to the formation of black and opaque colonies (**Supplementary Figure 1A**); eight isolates (IPR-4, IPR-5, IPR-6, IPR-7, IPR-8, IPR-10, IPR-11, and IPR-15) showed positive results for siderophore production, as confirmed by the orange halo around the colonies (**Supplementary Figure 1B**), whereas eight isolates (IPR-2, IPR-3, IPR-4, IPR-5, IPR-7, IPR-8, IPR-9, and IPR-16) showed positive test results for phosphate solubilization, as indicated by the solubilization of phosphates in the NBRIP medium, forming a clear zone along the colonies (**Supplementary Figure 1C**). Salkowski’s assay revealed that three isolates, IPR-4, IPR-8, and IPR-9, produced more IAA than the other isolates IPR-7 and IPR-12 with moderate pink color, whereas the others did not show any pink color (**Supplementary Figure 1D**). Based on EPS, siderophore, phosphate, and IAA production, the four best-performing isolates, IPR-4, IPR-7, IPR-8, and IPR-9, were further subjected to a 3-day drought stress assay using PEG at four different concentrations (0%, 5%, 10%, and 15%). IPR-4 showed high growth at 15% PEG stress with a cell density of 10^−8^ cells/mL (OD_600 nm_ = 0.411) compared with other isolates at 8 h (**Supplementary Figure 2A**), and it was further shown that isolate IPR-4 had superior drought stress tolerance over the other isolates over 16 h, 24 h, and 32 h (**Supplementary Figures 2B–D**). Therefore, isolate IPR-4 was further examined for molecular identification. The test result assigned isolate IPR-4 to the genus *Bacillus* sp. with 93% identity to others, viz. *B. safensis* FO-36bT (AF234854) on the same clad with 100%, *B. pumilus* ATCC 7061T (AY876289) 100%, *B. subtilis* NCDO 1769T (X60646) 100%, *Bacillus licheniformis* DSM 13T (X68416) 100%, *Bacillus halmapalus* DSM8723T (X76447) 82%, *Bacillus simplex* DSM 1321T (D78478) 100%, *Bacillus megaterium* IAM 13418T (D16273) 100%, *Bacillus benzoevorans* DSM 591T (D78311) 89%, and an outgroup *Paenibacillus polymyxa* IAM 13419T (D16276) 79.8% (**Supplementary Figure 3**).

### 3.2 *In-vitro* detection of organic acid and IAA production by isolate IPR-4

**Figure 1:**
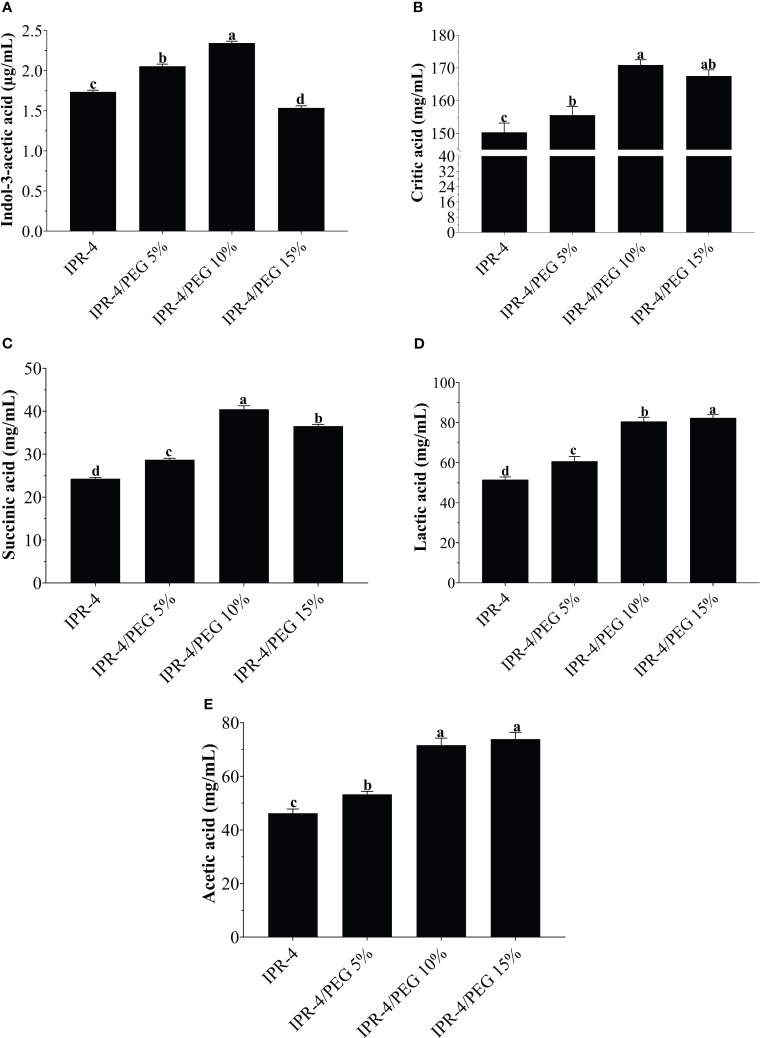
*In-vitro* quantification of Indol-3-acetic acid and organic acid production by the isolate IPR-4 under PEG stress, **(A)** Indol-3-acetic acid, **(B)** citric acid, **(C)** succinic acid, **(D)** lactic acid, and **(E)** acetic acid. Each data point represents the mean of the replicates; error bars represent standard errors at *p ≤*0.05. Lowercase letters represent the significant mean differences between replicates as evaluated by Duncan’s multiple range tests (DMRTs).

The GC/MS analysis results demonstrated that isolate IPR-4 exhibited a notable increase in IAA production, with a significant 24.9% increase under 5% PEG. In contrast, under 10% PEG, the increase in IAA production was only 18.5% compared with the control not detected (ND). However, at a high concentration of 15% PEG, the production of IAA decreased by 33.6% compared with the control, which contained only LB broth (**Figure 1A**). In the case of organic acids, HPLC test results indicate that isolate IPR-4 significantly increased the production of citric acid by 58% in 10% PEG and showed a decreased citric acid content by 8.1% in 15% PEG (**Figure 1B**). Succinic acid production was significantly increased by 61% in the concentration of 10% PEG (**Figure 1C**). In contrast, lactic acid and acetic acid production significantly increased by 43.6% and 50.7%, respectively, at 15% PEG concentrations (**Figures 1D, E**). The ANOVA result indicated significant (*p* ≤ 0.05) differences in organic acid production by isolate IPR-4 at different PEG concentrations.

### 3.3 Effect of IPR-4/MET co-inoculation on soybean growth and chlorophyll content under drought stress conditions

**Figure 2:**
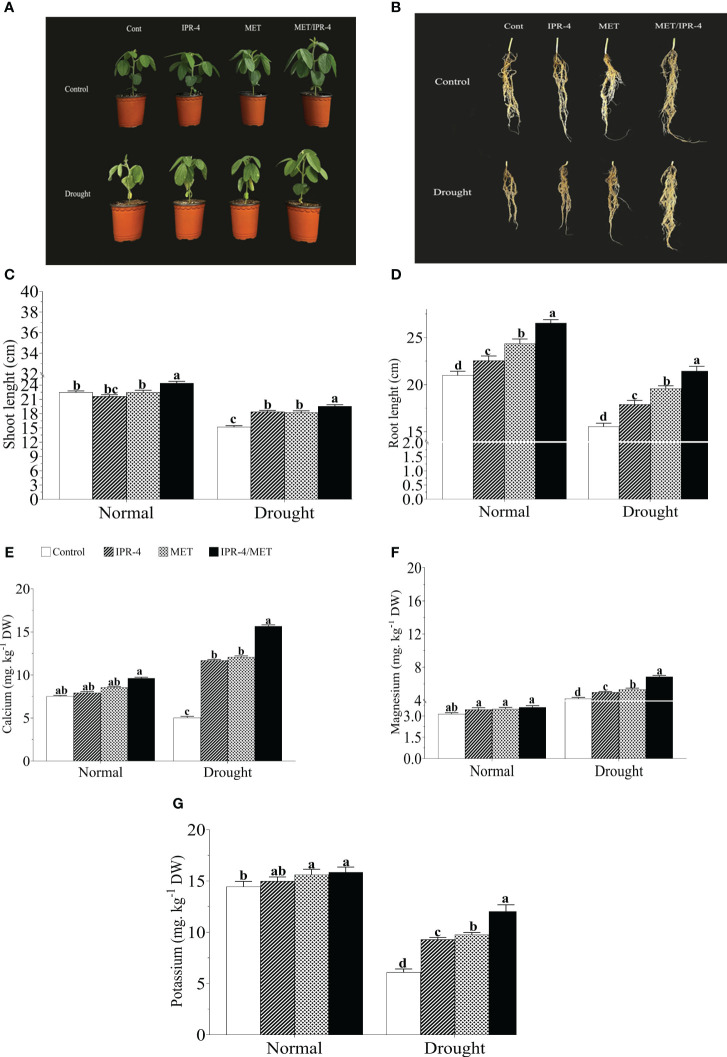
Effect of co-inoculation of IPR-4/melatonin (MET) on soybean growth parameters and nutrient mobilization during drought stress treatment: **(A)** shoot phenotype, **(B)** root phenotype, **(C)** shoot length, **(D)** root length nutrient mobilization, **(E)** calcium, **(F)** magnesium, and **(G)** potassium. Each data point represents the mean of triplicate measurements; error bars represent standard errors at *p ≤*0.05. Lowercase letters represent significant mean differences between replicates as evaluated by DMRTs.

The effects of isolate IPR-4 and melatonin on soybean plants under drought stress were assessed, as shown in **Table 1**. Co-inoculation of IPR-4/MET significantly increased soybean growth and biomass, whereas drought stress treatment resulted in a significant decrease in shoot length by 39.1%, root length by 46.7%, and chlorophyll content by 29.3% compared with control plants. Co-inoculation of isolate IPR-4/MET in drought-treated plants significantly increased shoot length by 33.3% compared with control plants. Under drought stress conditions, a significant reduction in plant height was observed in all treatments. However, the combined IPR-4/MET prevailed over IPR-4 and MET applied separately in alleviating the detrimental effects of drought stress (**Figures 2A, C**). Likewise, the most effective root growth-promoting ability was recorded in soybean plants treated with IPR-4/MET in response to drought stress (**Figures 2B, D**). Under the same conditions, plants grown under IPR-4/MET accumulated higher amounts of calcium (Ca^2+^, 50.7%; **Figure 2E**), magnesium (Mg^2+^, 31.2%; **Figure 2F**), and potassium (K^+^, 30.5%; **Figure 2G**). Unlike potassium (K^+^), a significant increase in Ca^2+^ and Mg^2+^ was recorded in plants treated with IPR-4 or MET alone or in combination in response to drought compared with normal growth conditions.

**Table 1:** Effects of IPR-4/MET co-inoculation on soybean growth.

Treatments	Shoot length (cm)	Root length (cm)	Chlorophyll (SPAD)
Control plants			
**Control**	22.5 ± 0.28b	21.0 ± 0.42d	40.8 ± 1.9ab
**IPR-4**	21.6 ± 0.35bc	22.5 ± 0.49c	41.5 ± 1.3ab
**MET**	22.5 ± 0.42b	24.3 ± 0.49b	41.4 ± 2.1ab
**IPR-4/MET**	24.4 ± 0.35a	26.5 ± 0.35a	43.3 ± 1.04a
Drought treated			
**Control**	15.2 ± 0.28c	15.5 ± 0.35d	28.5 ± 1.2c
**IPR-4**	18.4 ± 0.28b	17.9 ± 0.42c	32.3 ± 1.04b
**MET**	18.2 ± 0.35b	19.6 ± 0.28b	32.1 ± 0.96b
**IPR-4/MET**	19.6 ± 0.28a	21.4 ± 0.49a	37.07 ± 0.31a

Treatments are presented as control (distilled water only) and IPR-4 (isolate Bacillus sp.) MET melatonin and IPR-4/MET (co-inoculation of Bacillus. sp. and MET application). Data are the mean of triplicate readings. Different lowercase letters represent a significant difference evaluated by DMRT at p ≤0.05.

### 3.4 Effect of IPR-4 and melatonin application on ROS-scavenging activity

**Figure 3:**
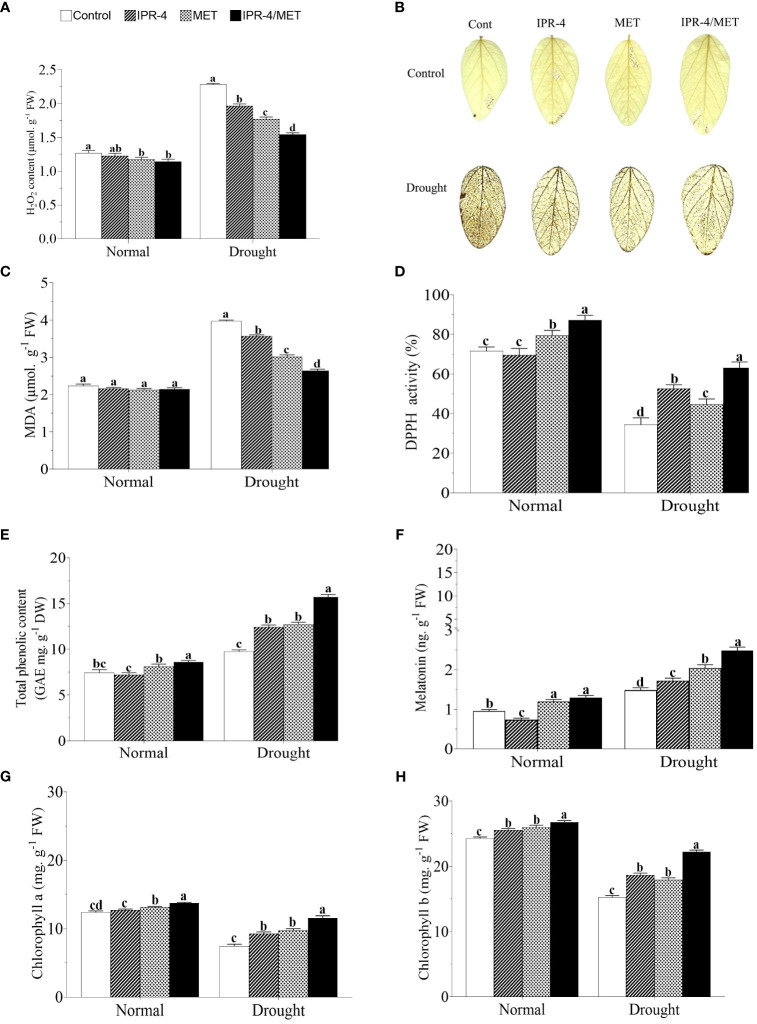
Effect of *Bacillus* sp. IPR-4 and MET on ROS, free radical scavenging, and chlorophyll contents in drought-treated soybean plants: **(A)** H_2_O_2_, **(B)** DAB staining for detecting hydrogen peroxide accumulation in the leaves, **(C)** DPPH, **(D)** malonaldehyde, **(E)** total phenolic contents, **(F)** melatonin, **(G)** chlorophyll a, and **(H)** chlorophyll b Each data point represents the mean of the replicates; error bars represent standard errors at *p ≤*0.05. Lowercase letters represent the significant mean differences between replicates as evaluated by DMRTs.

To assess the effects of IPR-4 and MET on soybean redox homeostasis, we measured H_2_O_2_ accumulation in drought-stressed plants. The results in **Figure 3A** revealed that drought stress significantly enhanced H_2_O_2_ production in soybean plants under normal conditions by 61.4%. However, the inoculation of IPR-4 or MET showed a reduction effect of H_2_O_2_ content of 18.3% and 16.7%, respectively. When co-inoculated with IPR-4/MET, H_2_O_2_ content decreased significantly (by 37.3%), a similar pattern observed under normal growth conditions. These results were consistent with the DAB staining shown in **Figure 3B**. Under the same conditions, malondialdehyde (MDA) content decreased significantly (by 30%) under IPR-4/MET co-inoculation compared to the control and when IPR-4 and MET were applied separately (**Figure 3C**).

### 3.5 Effect of IPR-4 and melatonin on free radical scavenging activity under drought stress conditions

The data in **Figure 3D** indicate that DPPH activity was significantly inhibited by 68% in sole-watered soybean plants exposed to drought stress. However, the inoculation of IPR-4 alone showed an increasing trend of DPPH activity, which was significantly enhanced by 38% under co-inoculation conditions. MET alone showed no significant difference in DPPH activity compared with the control treatment. In addition, the highest TPC was observed under IPR-4/MET co-inoculation in response to drought stress, followed by MET and IPR-4 alone compared to the slight increase in the control plants. It was also observed that the co-inoculation of IPR-4/MET significantly enhanced TPC content by 49.6% under normal growth conditions (**Figure 3E**). Similarly, MET content was higher in IPR-4/MET co-inoculated soybean plants under drought stress conditions compared to the control and IPR-4 or MET applied separately (**Figure 3F**). Furthermore, we investigated the changes in chlorophyll (Chl) accumulation under drought stress. As shown in **Figures 3G, H**, Chl a and b decreased significantly in sole-watered soybean plants exposed to drought stress, but an increasing pattern was observed when plants were inoculated with IPR-4 or when MET was applied. This effect was boosted when IPR-4/MET were co-inoculated.

### 3.6 Effect of IPR-4 and melatonin co-inoculation on endogenous ABA and SA

**Figure 4:**
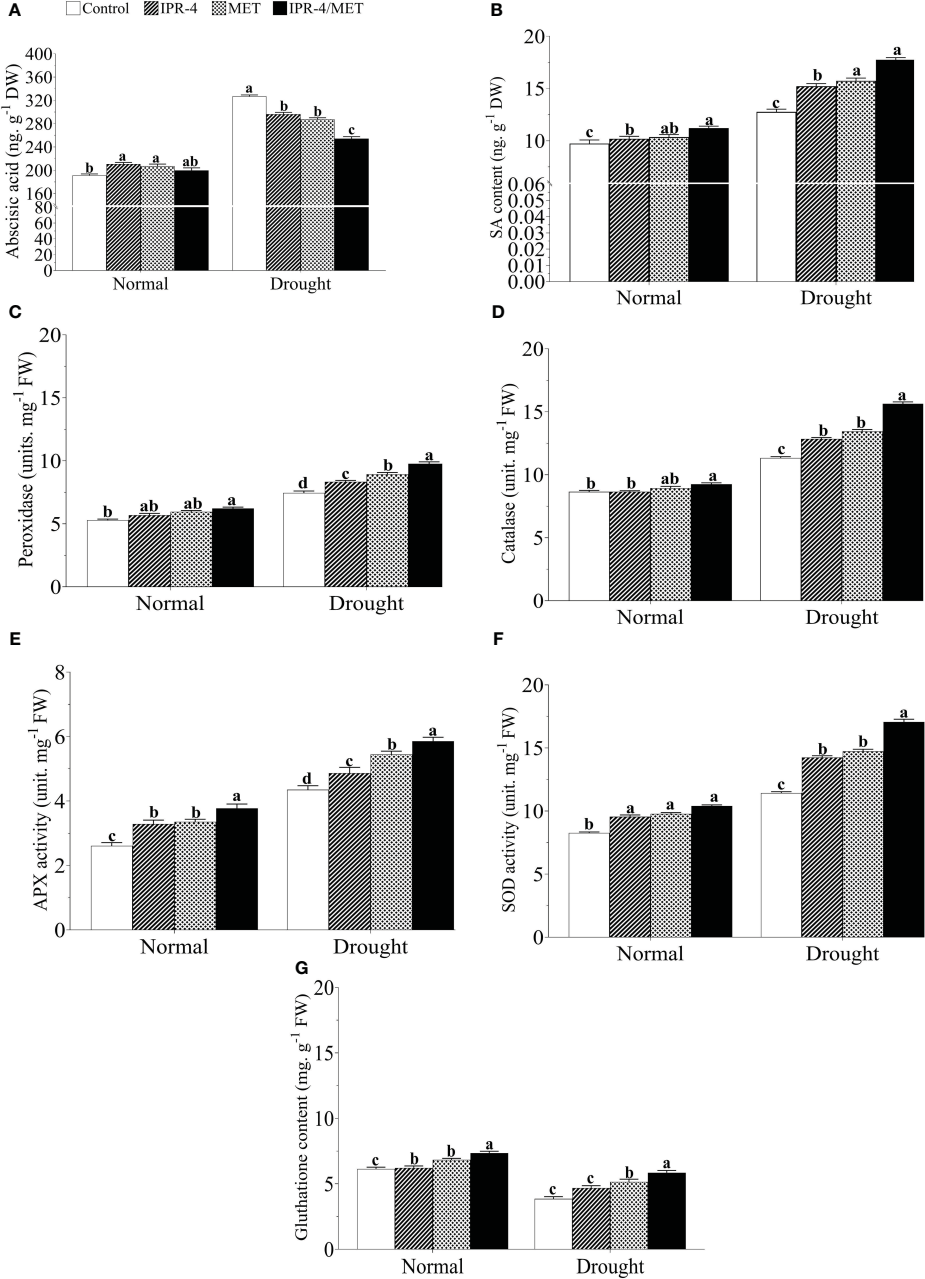
Effects of *Bacillus* sp. IPR-4 and MET on phytohormones and antioxidant activity in drought-treated soybean plants. **(A)** ABA, **(B)** SA, **(C)** peroxidase (POD), **(D)** catalase (CAT), **(E)** ascorbate peroxidase (APX), **(F)** superoxide dismutase (SOD), and **(G)** glutathione reductase (GSH). Each data point represents the mean of the replicates; error bars represent standard errors at *p ≤*0.05. Lowercase letters represent the significant mean differences between replicates as evaluated by DMRTs.

Drought stress caused a significant increase in ABA content in soybean plants for all treatments. The highest ABA level (88.9% increase) was recorded in drought-treated control plants compared with the normal growth conditions, whereas there was a decreasing pattern in IPR-4 and MET-treated plants, which reached its lowest level when IPR-4/MET were co-inoculated (25.5% decrease) (**Figure 4A**). Knowing that ABA antagonizes with other hormones such as SA, we also measured SA accumulation in soybean plants grown under IPR-4, MET, or IPR-4/MET and subjected to drought. As shown in **Figure 4B**, SA content increased in response to drought stress, but its accumulation was enhanced with IPR-4 inoculation and MET application and reached its highest level (29.1% increase) in IPR-4/MET co-inoculated soybean plants.

### 3.7 Effect of IPR-4 and melatonin on antioxidant enzyme activity under drought stress

The activity of the POD enzyme was measured in drought-treated plants earlier inoculated with IPR-4 or treated with MET alone or co-inoculated compared to that of control plants. As indicated in panel C of **Figure 4**, POD activity increased with drought stress in all treatments as expected but was further enhanced by the co-inoculated IPR-4/MET treatment (38.4% increase) compared with the control. The sole inoculation of IPR-4 or MET application also increased POD levels in drought-treated plants by 19.5%. In addition, **Figures 4D–F** show similar increasing patterns of CAT (34.14 increase), APX (76.8% increase), and SOD (69.8% increase) activities in response to drought stress, with the co-inoculated IPR-4/MET treatment having the highest enzymatic antioxidant activity promoting effect (39.8% increase in drought vs. control). Furthermore, a 36.7% GSH decrease was recorded in drought-stressed control plants, whereas the sole co-inoculation of IPR-4 or MET application resulted in a significant increase in GSH content and their co-inoculation boosted GSH content (31.6% increase) under the same conditions (**Figure 4G**).

### 3.8 Effect of IPR-4 and melatonin co-inoculation on drought stress response genes and transcription factors

**Figure 5:**
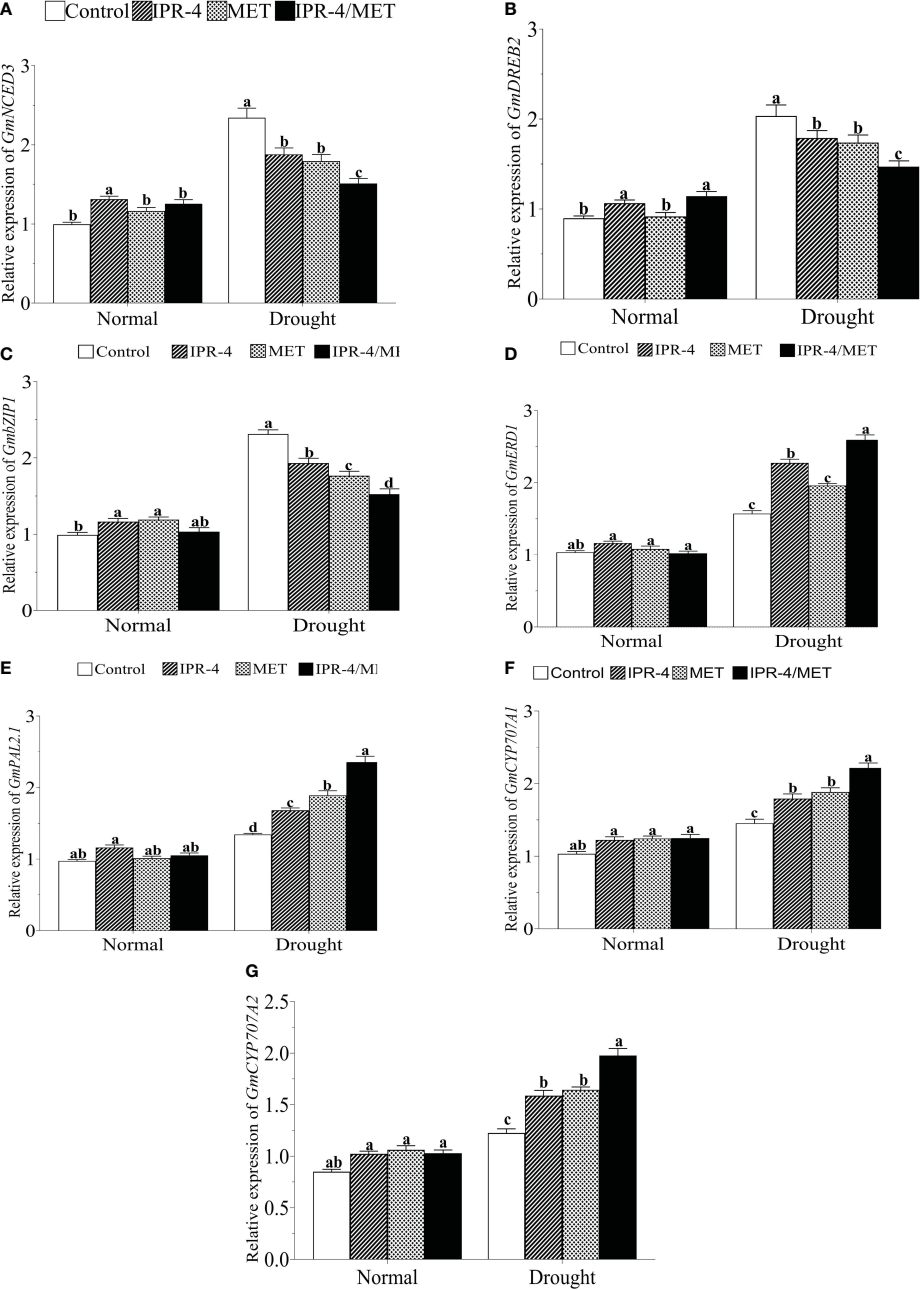
Effects of *Bacillus* sp. IPR-4 and MET co-inoculation on **(A)** *GmNCED3*, **(B)** *GmDREB2*, **(C)** *GmbZIP*, **(D)** *GmERD1*, **(E)** *GmPAL2.1*, **(F)** *GmCYP707A1*, and **(G)** *GmCYP707A2* on soybean under drought stress treatments. Each data point represents the mean of triplicate measurements; error bars represent standard errors at *p ≤*0.05. Lowercase letters represent significant mean differences between replicates as evaluated by DMRTs.

To further investigate the change in the transcript accumulation of drought marker genes, we analyzed the expression of well-established drought marker genes. The results in **Figure 5A** show that the expression of *GmNCED3* (ABA biosynthesis pathway gene) was significantly upregulated by drought stress in drought-stressed control plants, whereas a decreasing pattern was observed in the sole IPR-4- and MET-treated plants. However, the expression of *GmNCED3* showed no significant change under IPR-4/MET co-inoculation in response to drought stress compared to the normal growth conditions. Under the same conditions, *GmDREB2* (**Figure 5B**) and *GmbZIP1* (**Figure 5C**) exhibited similar transcript accumulation patterns with *GmNCED3*. In contrast, drought stress significantly upregulated the expression of *GmERD1* (**Figure 5D**), *GmPAL2.1* (**Figure 5E**), and *GmCYP770A1* (**Figure 5F**), and *GmCYP770A2* (**Figure 5G**) in a similar manner, with IPR-4/MET co-inoculation exerting the highest gene expression inducing effect, followed by the sole MET and IPR-4, except in *GmERD1* where IPR-4 inoculation prevailed over MET sole treatments (**Figure 5D**).

## 4 Discussion

### 4.1 Co-inoculated IPR-4/MET promotes plant growth and root development in soybean under drought stress

**Figure 6:**
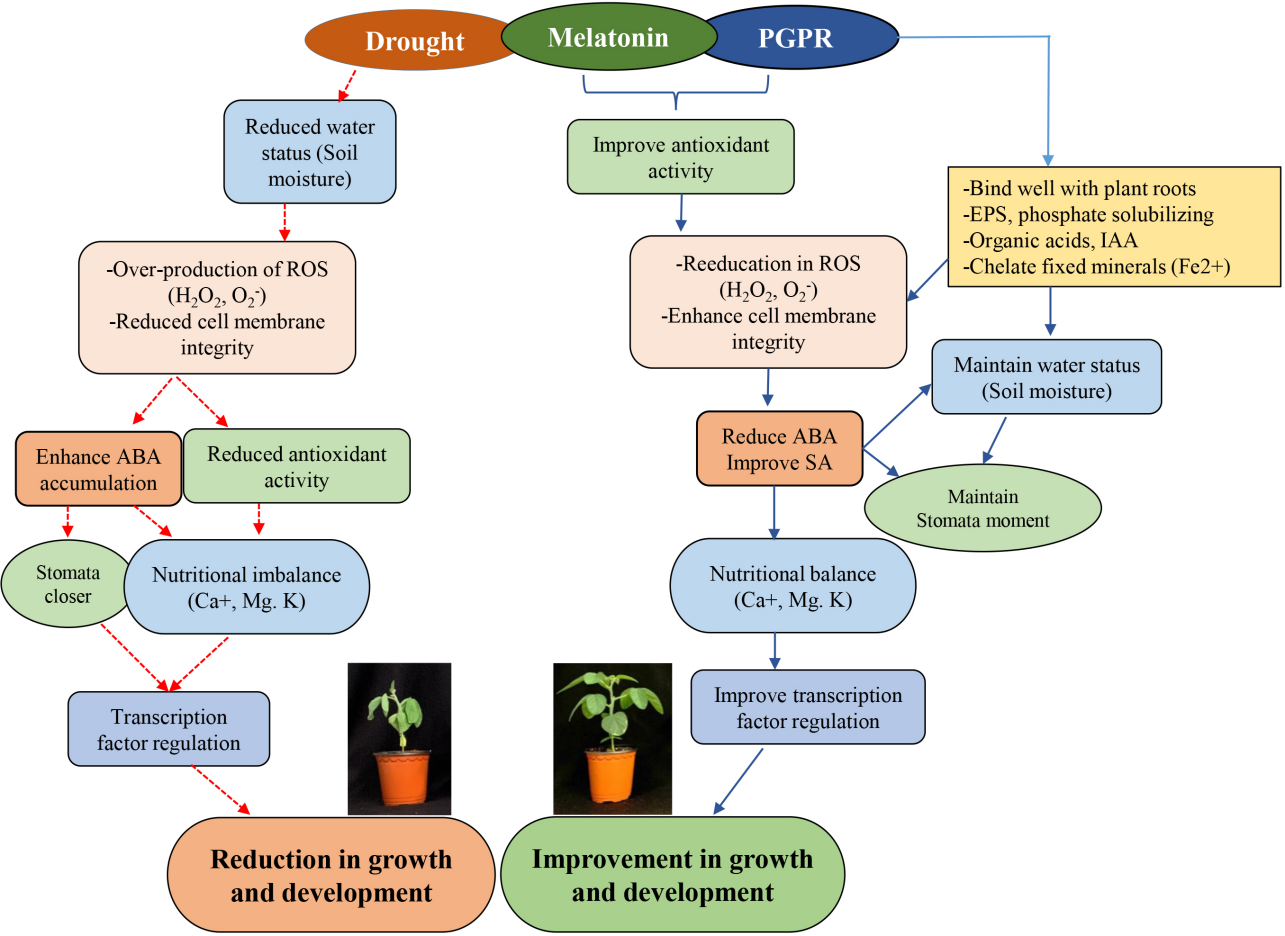
Schematic diagram illustrating synergy between Melatonin and Bacillus sp. IPR-4 in ameliorating drought stress tolerance in soybean via antioxidants, hormonal and transcription regulations.

Evidence of MET or plant growth-promoting rhizobacteria (PGPR) enhancing drought tolerance in plants is well-documented (Vurukonda et al., 2016). A study by Zhao et al. (2021) suggested that MET enhances drought tolerance in maize by regulating leaf stomatal conductance, carbon and nitrogen metabolism, and antioxidant and associated transcriptional machinery. Similarly, PGPRs, despite their established ability to promote growth and development, mediate plant resilience to abiotic stresses, including drought. It appears from the available reports that the mechanisms underlying MET- or PGPR-enhanced tolerance to abiotic stress activate similar enzymatic and non-enzymatic (Ahmad et al., 2022) antioxidant systems (Al-Turki and Murali, 2023) and influence the regulation of several stress-responsive genes. It is not clear whether these in-depth regulatory mechanisms occur directly or indirectly or generally in an uncoordinated manner at the whole-plant level; however, it has been established that these mechanisms involve changes in photosynthetic efficiency, phytohormone production and signaling, redox potential, or transcriptional regulation (Jahan et al., 2023). Furthermore, the synergetic effect of PGPR and other compounds has been reported. For instance, the application of 100 µM of MET was shown to improve drought stress via hormonal regulation and antioxidant systems when co-inoculated with the bacterium *Lysinibacillus fusiformis* (Imran et al., 2023). The *Bacillus* sp. has been proposed to have growth-promoting ability in plants (Dobrzyński et al., 2022). Our data suggest that co-inoculation of *Bacillus* sp. strain IPR-4/MET significantly enhanced plant growth and development under normal growth conditions. However, sole inoculation with the strain IPR-4 or MET showed a slight increase but a non-significant effect on plant growth. However, root development was significantly promoted under both sole application and co-inoculation, with the latter being the most effective. We further observed that the co-inoculation of IPR-4/MET alleviated the detrimental effect of drought stress induced by polyethylene glycol (PEG6000) in soybean, therefore prevailing over the sole inoculation of either IPR-4 or MET application and the control treatment as shown in **Figure 6**. *Bacillus* sp. strain IPR-4 showed a high survival rate under 15% PEG6000, which is much higher than that reported by Imran et al. (2023), where the bacterium *L. fusiformis* could only survive under 12% PEG6000. The efficiency of PGPR to ameliorate plants’ ability to avoid, escape, or tolerate abiotic stress events could be species-dependent and also depends on the duration of exposure to stress, in addition to the target genetic loci coupled with ROS scavenging and the antagonistic or synergetic relationship between phytohormones.

### 4.2 IPR-4/MET mediates nutrient solubilization and mobilization in plants

Rhizobacteria, such as *Bacillus aryabhattai* MS3, have a high siderophore-producing ability necessary to support plant growth under salinity conditions with low iron availability (Sultana et al., 2021). Siderophores, iron-binding compounds produced by certain microorganisms, including PGPR, facilitate iron uptake, strengthening the defense system of plants. A recent study by Wang et al. (2022) proposed that the siderophore bacterium *Paris polyphylla* var. *yunnanensis* could be a potential biofertilizer. It was interesting to see that *Bacillus* sp. strain IPR-4 exhibited a high ability to synthesize siderophores, which may explain its high iron mobilization from the soil as suggested earlier (Khasheii et al., 2021). Similarly, Zhang et al. (2022) found that *Bacillus pumilus* stimulates the production of 2-keto-L-gulonic acid in a vitamin C microbial fermentation system. *Bacillus pumilus* was also proposed by Dipta et al. (2017) to have a high phosphate-solubilizing potential for the enhancement of cauliflower (*Brassica oleracea* var. *botrytis* L.) root development. Here, we also observed that *Bacillus* sp. strain IPR-4 can solubilize insoluble phosphate (Ca (PO_4_)_2_) in the NBRIP medium into soluble phosphate ions (H_2_(PO_4_)^−^) and (H(PO_4_)^2−^). These results suggest that the enhanced phosphate use efficiency from poor soils through the improved rooting system contributed to the recorded drought stress alleviation responses.

Furthermore, studies support the production of bacterial EPS via sucrose assimilation, thus facilitating microbial adaptation (Fuso et al., 2023). In their study, Kang et al. (2009) reported a novel strain of *Acinetobacter calcoaceticus* that produces extracellular gibberellin (GA) and has a high potential for phosphate solubilization. Our data suggest a rapid production of EPS as soon as drought stress was induced (within 24 h) and gradually decreased with incubation time. This production is essential for microbial, metabolic, and physiological processes, including biofilm formation, microbial cell signaling and communication, and adhesion to surfaces (Fuso et al., 2023).

Moreover, the drought stress tolerance strategy involves the mobilization of various resources by plants which necessitates a high energy balance; sugars, amino acids, and organic acids play crucial roles in maintaining balanced plant growth and root development, mediating nutrient solubilization, supporting respiration, and enhancing photosynthetic efficiency (Xie et al., 2022). The recorded enhanced production of organic acids (citric acid, succinic acid, lactic acid, and acetic acid) by *Bacillus* sp. strain IPR-4 in response to PEG6000-induced drought stress was concentration-dependent. The release of organic acids by *Bacillus* sp. is hereby proposed to facilitate the chelation of cations bound to phosphate, as shown by the formation of a clear transparent ring zone around the bacterial colony in the phosphate-solubilizing medium. In a converse approach, Sweetman et al. (2014) observed an increase in malate concentration in *Vitis vinifera* when a high PEG concentration was applied. The accumulation of organic acids, in turn, leads to increased chlorophyll content and high photosynthetic efficiency (Vass et al., 2005).

Under the same conditions, our data showed that the co-inoculation of *Bacillus* sp. IPR-4 with MET enhanced the absorption of essential nutrients such as K^+^, Ca^2+^, and Mg^2+^, which suggests the existence of a synergistic interaction between *Bacillus* sp. strain IPR-4 and MET, resulting in improved nutrient mobilization. These events may help plants strengthen their intrinsic defense mechanisms during drought stress. However, it should be noted that K^+^ levels remained relatively low in soybean plants co-inoculated with *Bacillus* sp. strain IPR-4 compared to control plants (**Figure 6**).

### 4.3 IPR-4/MET regulates redox homeostasis and hormonal balance under drought stress

A balanced redox homeostasis is essential to maintain a balanced plant growth and development under changing environmental conditions, including drought stress. The latter causes plants to produce more ROS, which exerts a detrimental effect on plant growth and alters metabolic processes (Begum et al., 2022). The action of SOD is resumed in dismutating superoxide radicals (O_2_^•^), which generate H_2_O_2_ which is then reduced into water through the action of CAT enzymes and oxygen by GSH under drought stress conditions. Studies have shown ROS-scavenging attributes of certain PGPRs (Habib et al., 2016) or MET (Begum et al., 2022) applied solo. The recorded significant increase in SOD, CAT, APX, and POD is an indication of the degree of drought stress induction. Although the sole inoculation of *Bacillus* sp. IPR-4 or MET resulted in lower ROS levels and increased antioxidant activity, the co-inoculation of *Bacillus* sp. IPR-4 MET offers higher additive effects, higher ROS-scavenging ability, balanced redox potential, and improved photosynthetic efficiency of soybean plants subjected to drought stress, which resulted in a significant reduction in H_2_O_2_ accumulation and lipid peroxidation. Similarly, IPR-4 co-inoculated with MET resulted in low ABA levels, which antagonized SA accumulation under drought stress signaling and molecular responses in soybean plants under drought stress conditions. A study by Kuar et al. (2024) suggested that SA application enhances the transcript accumulation of *GmPAL2* in drought-stressed soybean plants.

### 4.4 IPR-4/MET-mediated drought tolerance involves drought marker transcription factors and ABA signaling

Inoculation of *Bacillus* spp. has been shown to alter the transcriptional regulation of several defense-related genes in plants (Samaniego-Gámez et al., 2023). *Bacillus* spp. are also identified as biostimulants, in which the biostimulation process occurs via phytohormone production (Mahapatra et al., 2022). In a recent study, Xu et al. (2022) proposed that *Bacillus subtilis*, a common PGPR, confers abiotic stress tolerance by induced systemic resistance (ISR), biofilm formation, and lipopeptide production. In a converse approach, the bacterium *B. aryabhattai* was shown to induce changes in the transcript accumulation of several genes to stimulate plant growth. Among all plant stress-related hormones, ABA prevails over other hormones when plants are exposed to abiotic stress (Muhammad Aslam et al., 2022). Under stress conditions, *NCED3* is a key player in ABA biosynthesis pathways that modulate stress response and activate downstream signaling toward stress tolerance (Barrero et al., 2006). Reports have suggested that the application of MET negatively affects ABA production in plants via the control of ABA biosynthesis genes and transcriptional reprogramming of stress-responsive genes, including cytochrome oxidase-encoding genes, such as *CYP707A1* and *CYP707A2* (Ali et al., 2023).

Drought-responsive transcription factors (TFs), such as the basic leucine zipper (bZIP) and dehydration response element binding (DREB) protein, are key regulators of drought tolerance in plants (Tao et al., 2024). In soybean, a member of the bZIP TF family, *GmbZIP15*, was shown to negatively regulate drought stress response (Zhang et al., 2020). Studies have indicated that melatonin application suppresses heat stress by altering ABA and cytokinin biosynthesis and signaling in perennial ryegrass (*Lolium perenne* L.) (Zhang et al., 2017) and downregulates *NCED3* and *bZIP*, resulting in increased CK accumulation in *Malus* spp (Li et al., 2015). and *Pinellia ternata* (Ma et al., 2020) under abiotic stress. It was interesting to see that the transcriptional levels of *GmNCED3*, *GmDREB2*, and *GmbZIP1* increased significantly in drought-stressed control plants, whereas a significant decrease was recorded, concomitant with the change in ABA content, especially in soybean plants co-inoculated with *Bacillus* sp. IPR-4 and MET. Under the same conditions, the transcript accumulation levels of *GmERD1*, *GmPAL2.1*, *CYP707A1*, and *A2* were higher in the combined treatment (co-inoculation of *Bacillus* sp. IPR-4 and MET) compared with the control and sole IPR-4 or MET treatments. We also observed that these expression levels followed the SA accumulation pattern. It could then be said that *Bacillus* sp. IPR-4 and MET-mediated regulation of *GmDREB2* and *GmbZIP1* would be ABA-dependent, similar to that of *GmCED3*, whereas that of *GmERD1*, *GmPAL2.1*, *CYP707A1*, and *A2* would be SA-dependent.

## 5 Conclusion

Drought stress is a major threat to soybean cultivation, thus inducing significant yield loss and quality. Sole inoculation with PGPR or MET has been investigated for its growth-promoting abilities and involvement in abiotic stress response mechanisms. This study investigated the ability of *Bacillus* sp. IPR-4 co-inoculated with MET to enhance soybean tolerance to drought. The results demonstrated the ability of the strain IPR-4 to colonize plants, in a synergistic relationship with MET, to promote plant growth and development and to alleviate the negative effects of drought stress. Herein, the mechanism underlying *Bacillus* sp. IPR-4/MET-mediated drought tolerance in soybean is proposed to involve key ROS-scavenging antioxidant enzymes (SOD, CAT, APX, GSH), coupled with the activation of an array of drought-responsive ABA-dependent *GmNCED3*, *GmbZIP1*, or *GmDREB2* TFs. However, *GmPAL2*, *GmERD1*, *GmCYP707A*, and *A2* were highly induced in *Bacillus* sp. IPR-4/MET co-inoculated plants, in a pattern similar to that of SA accumulation in response to drought stress, suggesting their possible dependence on SA rather than ABA signaling. In addition, the observed ability of *Bacillus* sp. IPR-4 to enhance macronutrient uptake, phosphate solubilization, and iron uptake mediated by siderophores may have significantly contributed to the adaptive response mechanism toward drought tolerance in soybean. Taken together, these results provide insights and improve our understanding of the mechanisms underlying the *Bacillus* sp. IPR-4/MET synergetic relationship to enhance plant drought stress tolerance. The *Bacillus* sp. IPR-4 co-inoculated with MET can then be used as an effective biostimulant to promote plant growth and enhance the resistance to drought stress in a sustainable manner.

## Data availability statement

The original contributions presented in the study are included in the article/**Supplementary Material**, further inquiries can be directed to the corresponding author/s.

## Author contributions

PO: Conceptualization, Data curation, Formal analysis, Investigation, Methodology, Visualization, Writing – original draft, Writing – review & editing. I-JL: Funding acquisition, Project administration, Resources, Supervision, Validation, Writing – review & editing. MI: Conceptualization, Software, Supervision, Validation, Writing – review & editing. SS: Writing – review & editing. S-MK: Supervision, Validation, Writing – review & editing. NKR: Writing – review & editing. CF: Data curation, Formal analysis, Writing – review & editing. ZD-D: Formal analysis, Writing – review & editing, Data curation. H-JG: Formal analysis, Writing – review & editing, Software. MI-UH: Formal analysis, Writing – review & editing. E-HK: Formal analysis, Writing – review & editing, Software. MNM: Writing – review & editing, Data curation, Formal analysis, Investigation. SB: Supervision, Validation, Writing – review & editing. WK: Supervision, Validation, Writing – review & editing.
