# Supplementary material for: Combined application of melatonin and Bacillus sp. strain IPR-4 ameliorates drought stress tolerance via hormonal, antioxidant, and physiomolecular signaling in soybean
Source: Front Plant Sci. 2024 Jun 21;15:1274964. doi: 10.3389/fpls.2024.1274964 (PMC11224487; doi:10.3389/fpls.2024.1274964)
Supplement: Supplementary file 1 [file DataSheet_1.pdf]

Combined application of melatonin and *Bacillus spp.* IPR-4 ameliorates drought stress tolerance via hormonal, antioxidant, and physio-molecular signaling in soybean

Peter Odongkara†[1] Muhammad Imran†[2]*, Shifa Shaffique[1], Sang-Mo Kang[1], Nkulu Kabange Rolly[1], Chebitok Felistus[1], Saqib Bilal[3], Zhao Dan-Dan[4], Md.Injamum-Ul-Hoque[1], Eunhae Kwon[1], Mohammad Nazree Mong[1], Ho-Jun Gam[1], Won-Chan-Kim[1], and In-Jung lee*[1]

[1]Department of Applied Biosciences, Kyungpook National University, Daegu 41566, Republic of Korea
[2]Biosafety Division, National Institute of Agricultural Sciences, Rural Development Administration, Jeonju 54874, Korea
[3]Natural and Medical Science Research Center, University of Nizwa, Nizwa 616, Oman
[4]Crop Foundation Research Division, National Institute of Crop Sciences, Rural Development Administration, Wonju 55365, Republic of Korea

[†]These authors equally contributed to this work.

*** Correspondence**: In-Jung Lee and Muhammad Imran
Email: ijlee@knu.ac.kr and m.imran02@yahoo.com

Table. S1: List of primer sequences used in real-time PCR

Gene		Forward
NCED3	F	5’- ACCACCTCTTCGACGGCGACGGAATGGT-3’
	R	5’- ATGGCGAGGAGTTTTCCGTTGAAGAAGA-3’
CYP707A1	F	5’- CAGGCTCAATGGGGTGGCCG-3’
	R	5’- AAAGCGTGCAGCCTCAGGGC-3’
CYP707A2	F	5’- GCCCCGAGGCTGCCAAGTTT-3’
	R	5’- CACGTCAGCACACTGGCGGT-3’
PAL2.1	F	5’- GAGTACCGGAGGCCCGTCGT-3’
	R	5’- CACCGAACCCGGTGGTGACG-3’
GmDREB2	F	5’-GTTTTGGAATTGAGACAGGC-3’
	R	5’-ACCAACCATTTGACATAACG-3’
GmbZIP1	F	5’- ACCAACACCAACAACATCCA-3’
	R	5’- TTGAATGCTCAGCAGCAACT-3’
GmERD1	F	5’-CGTCCAGAATTGCTCAACAG-3’
	R	5’-TGGGGTTATAGCCTTGTTGG-3’
ACT11	F	5’- ATCTTGACTGAGCGTGGTTATTCC -3’
	R	5’- GCTGGTCCTGGCTGTCTCC -3’

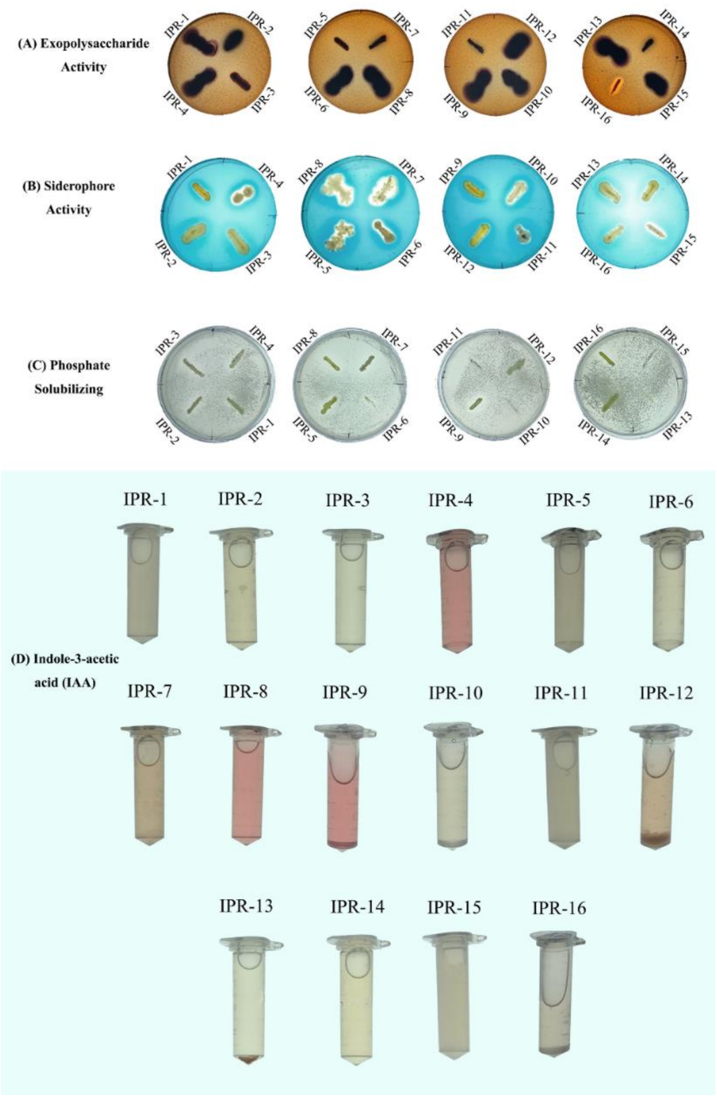

Fig. S1. Screening of isolates for PGPR traits: (A) EPS, (B) siderophore production, (C) phosphate solubilization, and (D) IAA production

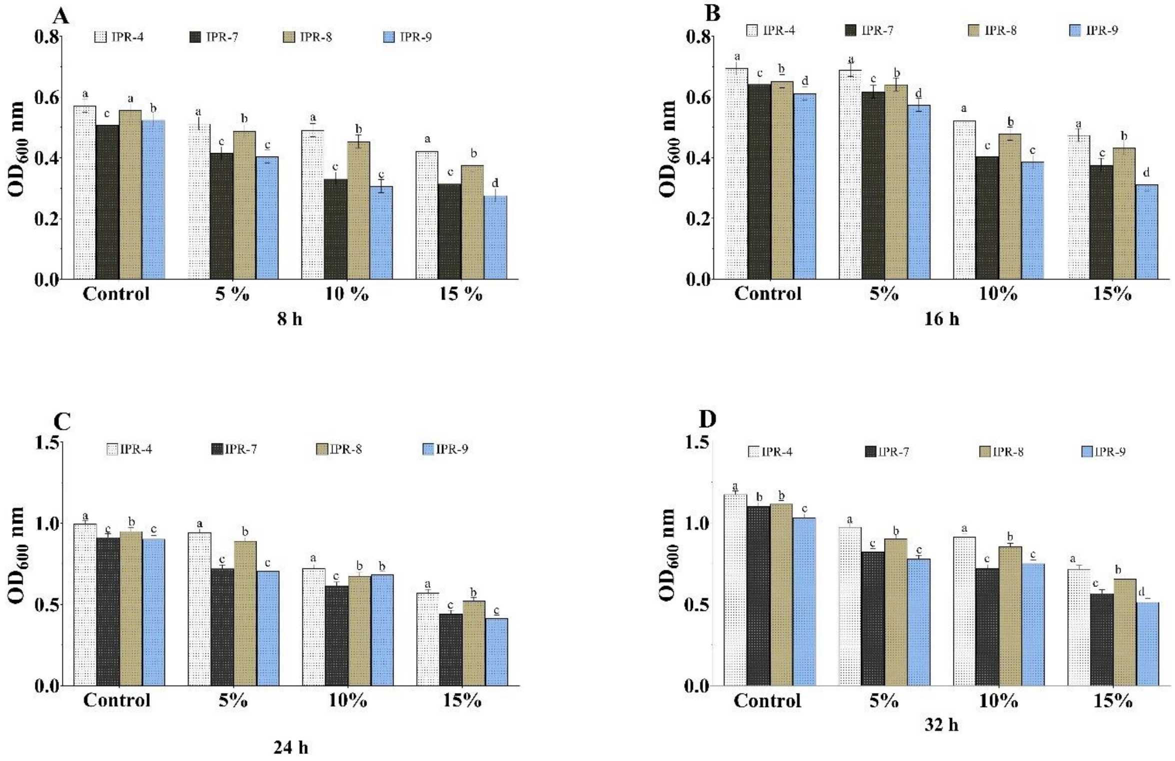

Fig. S2. Growth of isolates in different PEG concentrations (control, nutrient broth only 0 %) PEG, 5 % PEG, 10 % PEG, and 15 % PEG) at different time points (**A**) OD of isolates at 8 h, (**B**) OD of isolates at 16 h, (**C**) OD of isolates at 24 h, and (**D**) OD of isolates at 32 h.

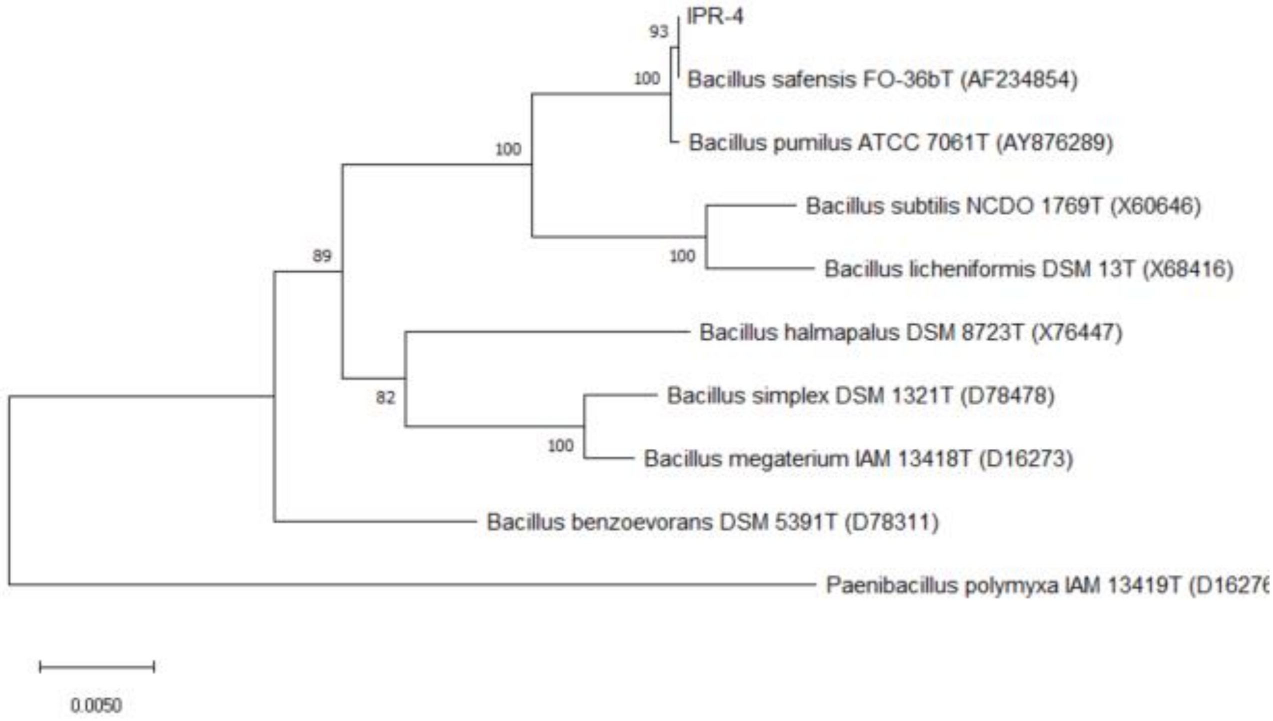

Fig. S3. A phylogenetic tree showing the evolutionary relationships of strain IPR-4 was constructed using the neighbor-joining method (Saitou and Nei, 1987). The neighbor-joining test was performed using 16S rRNA sequences obtained using NCBI BLAST. *Bacillus sp.* strain IPR-4 (acc. no. OP303352.1), *B. safensis FO-36bT (AF234854), B. pumilus* ATCC 7061T (AY876289), *B. subtilis NCDO 1769T (X60646), B. licheniformis* DSM 13T (X68416), *Bacillus halmapalus* DSM8723T (X76447), *Bacillus simplex* DSM 1321T (D78478)*, B. megaterium IAM 13418T (D16273), B. benzoevorans* DSM 591T (D78311), *and P. polymyxa* IAM 13419T (D16276). The optimal tree is shown. The percentage of replicate trees in which the associated taxa clustered in the bootstrap test (1000 replicates) is shown below the branches (Felsenstein, 1985). The tree is drawn to scale, with branch lengths in the same units as those of the evolutionary distances used to infer the phylogenetic tree. The analysis involved 10 nucleotide sequences. All ambiguous positions were removed for each sequence pair (pairwise deletion option). There were 1421 positions in the final dataset. Evolutionary analyses were conducted on mega 11 (Tamura et al., 2021).
