# Supplementary figures and images for: Combined application of melatonin and Bacillus sp. strain IPR-4 ameliorates drought stress tolerance via hormonal, antioxidant, and physiomolecular signaling in soybean
Source: Front Plant Sci. 2024 Jun 21;15:1274964. doi: 10.3389/fpls.2024.1274964 (PMC11224487; doi:10.3389/fpls.2024.1274964)

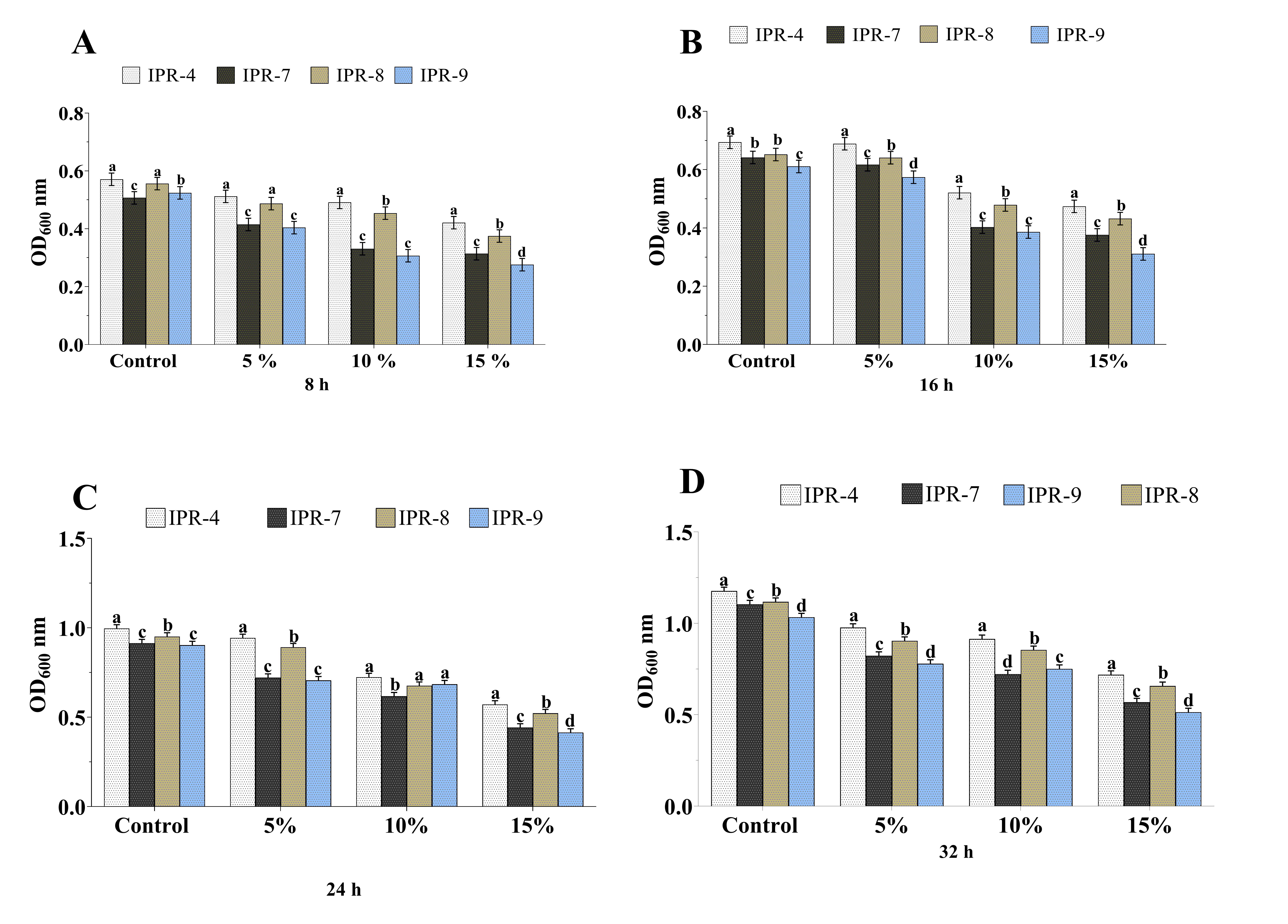

Supplement: Supplementary file 2 [file Image_1.jpeg]
